# Implementing EMI in Chinese music classes: Students’ perceived benefits and challenges

**DOI:** 10.3389/fpsyg.2023.1086392

**Published:** 2023-03-14

**Authors:** Pengcheng Su, Jiayin Kong

**Affiliations:** ^1^School of Liberal Arts, Metharath University, Pathum Thani, Thailand; ^2^Faculty of Education, Pathumthani University, Pathum Thani, Thailand

**Keywords:** English as a medium of instruction (EMI), benefits, challenges, English literacy, Chinese students, music classes

## Abstract

Recognizing the opportunities and problems of using English as a medium of instruction (EMI) enables teachers, students, and educational administrators to capitalize on the opportunities and mitigate the problems. Considering this, many researchers worldwide have explored the opportunities and problems of EMI courses. Yet, the advantages and disadvantages of implementing EMI in Chinese academic contexts have rarely been investigated. To fill this gap, the present research evaluated the benefits and challenges of implementing EMI in Chinese music classes. To accomplish this, a researcher-made scale was distributed among 74 Chinese music students. The thematic analysis of participants’ responses revealed that using English as a means of teaching and learning benefited Chinese music students in some ways. However, as the results of the thematic analysis indicated, Chinese music students experienced some serious challenges in EMI courses because of their limited English proficiency. Finally, the limitations, pedagogical implications, and future research directions are thoroughly explained.

## Introduction

In the past 10 years, with the globalization of education, an increasing number of Chinese students majoring in music have decided to go abroad for their further study (Lin and Lei, 2021). However, due to their limited English proficiency, they commonly experience various linguistic difficulties in non-Chinese educational settings where course content is mostly delivered in English (Fu and Wang, 2022). To improve Chinese students’ English proficiency, several music institutes in China have started using English as the medium of instruction (EMI). The penetration of the EMI phenomenon in all educational settings, including music classes, signals a rapid change from learning English as a foreign or second language to learning different academic subjects through English (Aizawa and Rose, 2020). EMI, as Macaro (2018) mentioned, has to do with “the use of the English language to teach academic subjects (other than English) in countries where the first language of the majority of the population is not English” (p. 19). Accordingly, the main goal of an EMI course is content learning, although language acquisition may be the implied purpose (Hu and Li, 2017). In EMI courses, the delivery of academic content, appraisal of learning outcomes, and group discussions are all conducted in English (Xu, 2017; Macaro et al., 2018; Macaro, 2019; Derakhshan, 2021).

Using English as the means of teaching and learning in Chinese music institutes is likely to bring some serious challenges for students since they typically attend EMI courses with limited English proficiency (Yang, 2017; Jiang et al., 2019; Hu and Wu, 2020). However, the implementation of EMI in these institutes may provide students with some precious opportunities as well (Zhao and Dixon, 2017; Curle and Derakhshan, 2021). Among them, one can refer to increased English proficiency and enhanced learning motivation as the most prominent advantages of EMI classrooms (Lei and Hu, 2014; Rose et al., 2020). As put forward by many educational researchers (Tong and Tang, 2017; Jiang and Zhang, 2019; Curle et al., 2020), knowing the benefits and challenges of using English as a means of education is critical in that it helps teachers and administrators improve the quality and efficiency of EMI classes. Thus, to enhance the efficiency of EMI courses in Chinese music institutes, their potential challenges and opportunities should be identified.

Given that understanding the challenges and opportunities of EMI courses improves their quality (Walkinshaw et al., 2017; Wei et al., 2017), many academics and researchers worldwide have investigated the advantages and disadvantages of implementing EMI in higher educational contexts (e.g., Bradford, 2016; Martínez, 2016; Cankaya, 2017; Kim, 2017; Kim et al., 2018; Phuong and Nguyen, 2019; Aizawa and McKinley, 2020; Toh, 2020; Diezmas and Barrera, 2021; Kamaşak et al., 2021; Soruç et al., 2021; Ter-Vardanyan, 2021; Wang and Derakhshan, 2023, to cite a few). Moreover, some scholars have particularly scrutinized the advantages and disadvantages of implementing EMI in Chinese academic settings (e.g., Yang et al., 2019; Pun and Thomas, 2020; Pun and Jin, 2021; Zhou et al., 2022, among others). However, to the best of the scholars’ knowledge, no empirical study has been undertaken to identify the opportunities and challenges of executing EMI in Chinese art and music classes. Against this backdrop, the current qualitative study intends to uncover the opportunities and challenges that Chinese music students typically experience in EMI courses.

## Literature review

English as the medium of instruction is a subset of content and language integrated learning (CLIL), which pertains to teaching academic subjects such as art, geography, history, and science through a foreign language (Dalton, 2011; Harrop, 2012). Simply said, CLIL is a pedagogical approach through which students learn the course content and a new language at the same time (Arnó-Macià and Mancho-Barés, 2015; Morton, 2018). In light of CLIL’s definition, Macaro (2018) defined EMI as the employment of the English language to instruct various academic subjects in regions where English is not the mother tongue (L1) of the majority of the population. In a similar vein, the “Japanese Ministry of Education, Culture, Sports, Science and Technology (MEXT)” conceptualized EMI as “courses conducted entirely in English, excluding those whose primary purpose is language education” (Rose et al., 2020, p. 2,150).

As noted by Nguyen et al. (2017), implementing EMI in educational environments may entail some important challenges and opportunities for both teachers and students, which need to be discovered. In this respect, several researchers have explored the problems and benefits of implementing EMI in different academic contexts, notably higher education contexts (e.g., Goodman, 2014; Hu, 2019; Phuong and Nguyen, 2019; Yang et al., 2019; Aizawa and McKinley, 2020; Drljaca and Vodopija-Krstanovic, 2020; Pun and Thomas, 2020; Toh, 2020; Derakhshan et al., 2021; Kamaşak et al., 2021; Pun and Jin, 2021; Soruç et al., 2021; Ter-Vardanyan, 2021; Zare and Derakhshan, 2021; Jones et al., 2022; Zhou et al., 2022, among others). As to the challenges of EMI classes, Yang et al. (2019), for instance, examined the challenges of implementing EMI in medical courses in China. Using surveys and group discussions, the researchers found that implementing EMI in Chinese medical courses brings four important challenges for teachers and students, namely unsatisfactory instruction, inappropriate instructional materials, limited classroom interactions, and teachers’ inability to instruct medical humanities. In a similar vein, Pun and Thomas (2020) explored the challenges and problems of executing EMI in different schools in Hong Kong. To do so, 19 teachers instructing chemistry, biology, and physics through English were interviewed. The analysis of participants’ answers revealed that executing EMI in Chinese schools brings various linguistic challenges (e.g., inability to teach and illustrate scientific ideas in English, inability to offer corrective feedback in English, etc.) for teachers. Later, in a quantitative inquiry, Kamaşak et al. (2021) investigated the language-related problems that Turkish university students face in EMI classes. To accomplish this, some online questionnaires were administered to 498 Turkish undergraduate students. The results demonstrated that speaking and writing were the most challenging skills for Turkish students.

Besides, as to the benefits and opportunities of EMI classes, Phuong and Nguyen (2019) examined Vietnamese students’ perceptions regarding the advantages of implementing EMI in business and information technology classes. To do this, a researcher-made questionnaire was distributed among 136 Vietnamese students majoring in business and information technology. The results showed that teaching course content in English considerably improves the English literacy of students, makes them more active and outgoing, and helps them find learning materials written in English. By the same token, Derakhshan et al. (2021) studied the advantages and opportunities of implementing EMI courses in Iran. In doing so, 24 EMI students and instructors of different nationalities were asked to complete an open-ended scale. Participants’ responses to the open-ended scale were analyzed using content analysis (CA). The analysis of responses resulted in some important themes, including “more access to specialized sources,” “more chances to improve English proficiency,” and “higher chance of continuing education abroad.”

Despite such attempts (e.g., Phuong and Nguyen, 2019; Yang et al., 2019; Pun and Thomas, 2020; Derakhshan et al., 2021; Graham and Eslami, 2021; Kamaşak et al., 2021; Gao et al., 2022; Wang, 2023), the advantages and disadvantages of executing EMI in Asian countries, including China, are not widely known. Put differently, a limited number of investigations have been undertaken to uncover the opportunities and challenges that Asian students experience in EMI courses. Considering this, the present inquiry sought to assess the benefits and challenges of implementing EMI in Chinese music classes. To do so, the following research questions were formulated:

▪ What are the Chinese music students’ perceptions towards the opportunities/benefits of the EMI courses?
▪ What are the Chinese music students’ perceptions towards the challenges/problems of the EMI courses?

## Methods

### Participants

Through the opportunity sampling strategy, also called convenience sampling, 74 music students were recruited from different classes at a private music institute in China. As noted by Dörnyei and Csizér (2012), the opportunity sampling strategy is a subset of non-probability sampling methods through which “members of the target population are selected only if they meet certain practical criteria, such as geographical proximity, availability at a certain time, or easy accessibility” (p. 82). In order to enhance the representativeness of the outcomes (Nassaji, 2020), the participants were selected from different age groups, gender (*male* and *female*), and educational levels (*freshman*, *sophomore*, *junior*, and *senior*). All students were assured that their personal data would be kept strictly confidential.

### Instrument

An open-ended scale (Appendix A) with two separate parts was used to find out the benefits and challenges of implementing EMI in Chinese music classes. Given the adequate English proficiency of participants, the questionnaires’ items were written in English. In the first part, participants were invited to enter their personal information, including age, gender, nationality, and level of education. In the second part, they were asked to respond to five open-ended questions regarding the implementation of EMI courses in Chinese music classes. Open-ended scales are commonly preferred to other data-gathering instruments as “they are easier to administer (notably when conducted online), offer more time to participants to fill out the questionnaires, and do not need to be transcribed” (Friedman, 2012, p. 189).

Prior to being distributed among participants, the open-ended scale was administered to seven non-participants. In light of the piloting outcomes, all items of the open-ended questionnaire were revised. The questionnaire items were then rigorously reviewed by five specialists who published several papers on EMI classes and their relevant issues. They checked the questionnaire items in terms of form and language. The final version of the open-ended questionnaire was produced with their sensible remarks in mind.

### Data collection procedure

At the very beginning, the E-version of the consent form was delivered to participants *via* WeChat messenger. Having received the written agreements, the researcher-made questionnaire was sent to participants. To receive more accurate responses (Rolfe, 2006), the participants received some brief explanations regarding the completion of the questionnaire. All surveys were answered in English and returned within a week.

### Data analysis

**Figure 1:**
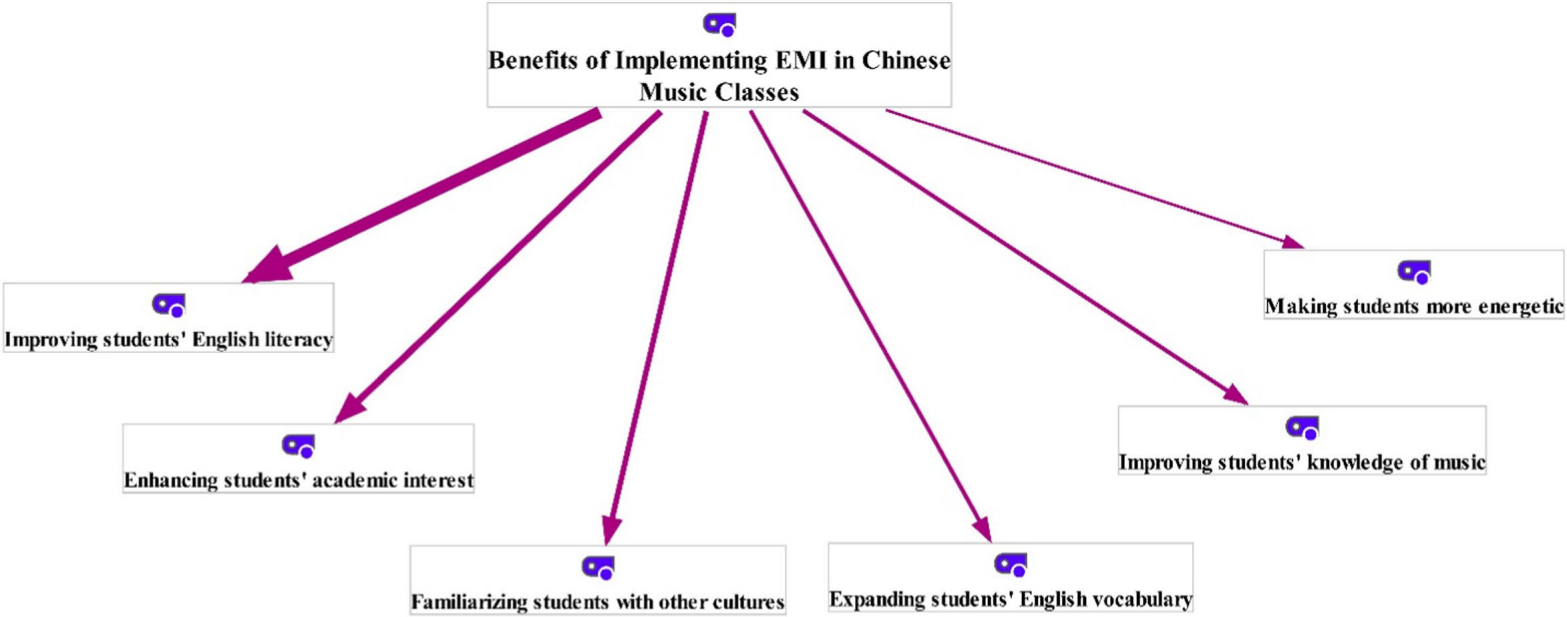
Benefits of implementing English as a medium of instruction (EMI) in Chinese music classes (the thicker the line, the more recurrent the theme).

**Figure 2:**
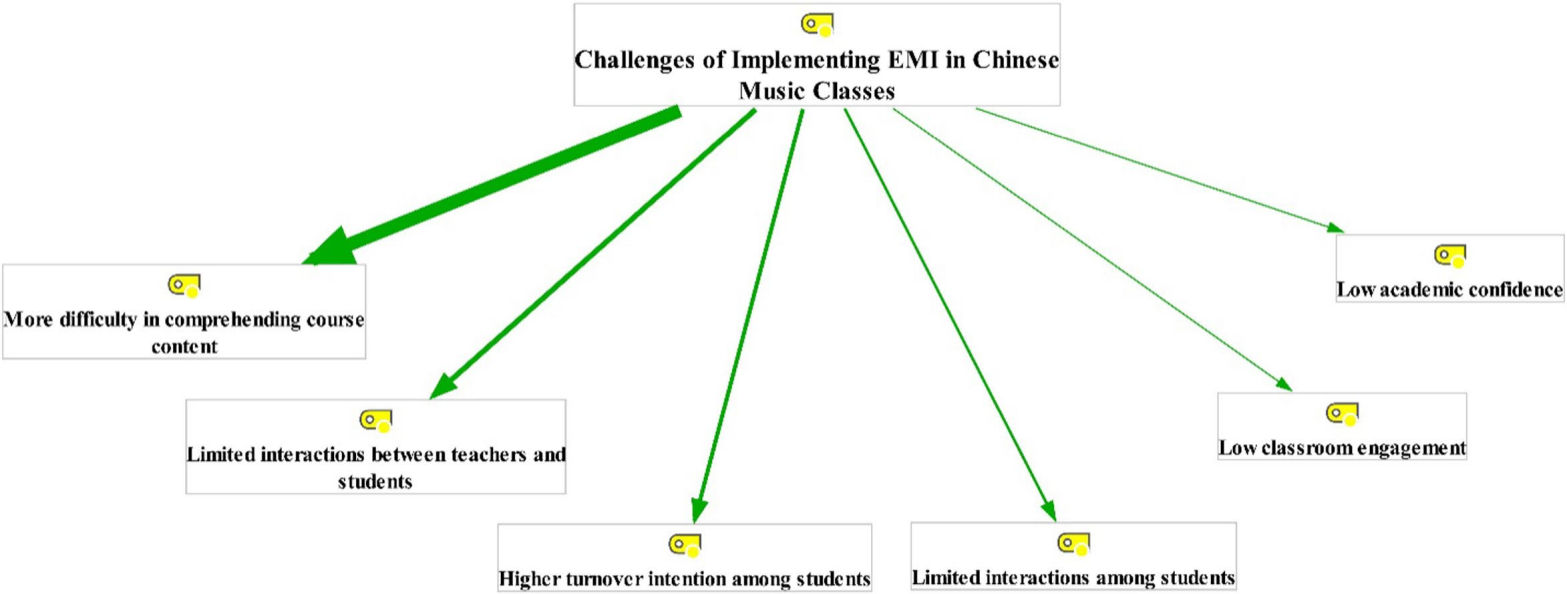
Challenges of implementing English as a medium of instruction (EMI) in Chinese music classes (the thicker the line, the more recurrent the theme).

The responses of Chinese music students to the open-ended scale were thematically analyzed using the last version of MAXQDA software (Version 2022). The rationale of employing MAXQDA software in this research was that “using a Computer-Assisted Qualitative Data Analysis Software (CAQDAS) can improve the credibility of the coding process” (Baralt, 2012, p. 228). To do so, the Gao and Zhang (2020) thematic analysis approach, which consists of five major phases, was used. To enhance the credibility of the extracted themes, all phases of this approach were simultaneously implemented by two analysts (the researcher and one of his colleagues). First, in the cleaning stage, the analysts looked through the gathered answers to locate and remove duplicates or irrelevant items. Following that, in the coding phase, they generated some tentative codes by rereading the collected answers. Then, in the phase of generating themes, the analysts grouped the tentative codes into some meaningful themes. As a result, two overarching themes and 12 sub-themes were generated (see Figures 1 and 2). Subsequently, in the categorization stage, the produced themes and sub-themes were categorized into higher-order umbrella terms. In the final stage of thematic analysis, producing the report, a summary of the whole process was prepared by the analysts. As pinpointed by Birt et al. (2016), “member checking enhances the trustworthiness of qualitative findings” (p. 1,803). Because of this, some of the participants were invited to check the relevance and accuracy of the produced themes and subthemes. Having assessed the accuracy of the produced themes and subthemes, the agreement between the two analysts was computed using Krippendorff’s alpha. The Krippendorff’s alpha (*α*) was measured to be 0.97.

## Findings

The outcomes of the current study illustrate the major benefits and challenges of implementing EMI in Chinese music classes. Accordingly, Chinese music students’ perceptions towards EMI implementation were grouped under two overarching patterns: (І) the benefits of implementing EMI in Chinese music classes and (П) the challenges of implementing EMI in Chinese music classes.

i. Benefits of Implementing EMI in Chinese Music Classes.

The participants were initially asked about the benefits of executing EMI in Chinese music classes. The examination of their answers resulted in six themes, including *improving students’ English literacy*, *enhancing students’ academic interest*, *familiarizing students with other cultures*, *expanding students’ English vocabulary*, *improving students’ knowledge of music*, and *making students more energetic* (Figure 1).

As demonstrated in Figure 1, the most frequent benefit mentioned by Chinese music students was that EMI courses significantly improve their English literacy. In fact, most of the participants believed that EMI courses help music students improve their English language skills, including reading, writing, listening, and speaking. For instance, some of the Chinese music students articulated that,

**Participant 3:** *I can practice speaking English when I answer questions in the process of learning.*

**Participant 7:** *Learning music through English can raise our English proficiency level.*

**Participant 13:** *To me, the most important advantage of EMI courses is that teaching course content through English improves students’ basic knowledge of English.*

**Participant 18:** *I think using English as the means of instruction can considerably enhance my English abilities.*

**Participant 21:** *I can improve my English language abilities while learning the course content.*

Another benefit of EMI classes noted by the majority of participants was that using English in music classes can increase the academic interest of students. They mentioned that learning a new language while mastering the course content can make students more enthusiastic and passionate about the learning process. To illustrate,

**Participant 24:** *Using English as a medium of instruction makes us more interested in learning music.*

**Participant 29:** *Learning course content through English can stimulate students’ academic interest.*

**Participant 31:** *Using EMI in music classes can cultivate students’ moral sentiments and stimulate their interest in learning.*

**Participant 43:** *Learning a new language makes EMI students more passionate about the learning process.*

As for the third benefit of EMI courses, participants referred to the role of EMI in familiarizing students with other cultures. The following statements clearly represent this advantage of EMI classes:

**Participant 4:** *Through learning music in English, we can get in touch with some western cultures and broaden our horizons.*

**Participant 6:** *Teaching and learning course content through English enables us to become familiar with the culture of English-speaking countries.*

**Participant 14:** *EMI classes help students have a better grasp of the cultural values of other nations.*

As to the other benefits of implementing EMI in music classes, participants mentioned the pivotal role of EMI in expanding students’ English vocabulary, improving students’ knowledge of music, and making students more energetic. The following sentences illustrate these benefits, respectively:

**Participant 27:** *Using English in music classes is of great help to students to increase their knowledge of English vocabulary.*

**Participant 33:** *It can also improve my understanding of various aspects of music knowledge.*

**Participant 41:** *Learning music and a new language concurrently may make students more energetic.*

ii. Challenges of Implementing EMI in Chinese Music Classes.

The participants were then questioned about the challenges and problems of implementing EMI in Chinese music courses. The evaluation of their responses culminated in six themes, including *more difficulty in comprehending course content*, *limited interactions between teachers and students*, *higher turnover intention among students*, *limited interactions among students*, *low classroom engagement*, and *low academic confidence* (Figure 2).

The most recurrent theme mentioned by Chinese students as the challenge of employing English in music classes is students’ difficulty in comprehending course content. Most of the participants maintained that teaching the academic subjects through English makes it difficult for students to understand the course content. This challenge is illustrated by the excerpts below:

**Participant 12:** *A disadvantage of using English in music courses is that students with poor English proficiency cannot fully understand the learning content.*

**Participant 15:** *Students who do not have a good command of English cannot easily comprehend the course content and keep up with the pace of the class.*

**Participant 51:** *Students can hardly understand the content of the classroom.*

**Participant 63:** *Almost all students struggle to comprehend the teachers’ instructions.*

Many students also mentioned limited classroom interactions as another serious challenge they typically experience in EMI courses. They noted that due to low English literacy, they are unable to effectively communicate with their teachers. The following excerpts can help to clarify the issue:

**Participant 50:** *Some students are slow to react and can’t effectively communicate with their teachers.*

**Participant 59:** *Due to limited English proficiency, students are unable to easily interact with their instructors.*

**Participant 68:** *It is burdensome for music students to interact with their teachers in English.*

Concerning the third challenge of EMI classes, participants mentioned the direct impact of these courses on students’ turnover intention. To illustrate,

**Participant 20:** *Using English as a medium of teaching leads students to leave the music classes.*

**Participant 28:** *Teaching academic subjects through English may discourage students from continuing the learning process.*

**Participant 73:** *Using English to teach music prompts students to leave the classes.*

Other significant issues raised by music students about EMI courses include limited student interaction, low classroom engagement, and low academic confidence. These issues are presented in the following statements, respectively:

**Participant 17:** *Most of the music students struggle to communicate with each other through English.*

**Participant 66:** *Due to limited English literacy, students are reluctant to engage in classroom activities.*

**Participant 74:** *Poor English proficiency gradually decreases students’ self-confidence in academic settings.*

## Discussion

The current qualitative study was designed to uncover the benefits and challenges of implementing EMI in Chinese music classes. Simply said, it aimed to illustrate how EMI classes are advantageous and disadvantageous for Chinese music students. As to the benefits or advantages of EMI courses, the outcomes of the thematic analysis revealed that the benefits of using English in Chinese music classes can be grouped into six unique themes: *improving students’ English literacy*, *enhancing students’ academic interest*, *familiarizing students with other cultures*, *expanding students’ English vocabulary*, *improving students’ knowledge of music*, and *making students more energetic*. As shown in Figure 1, most of the music students asserted that executing EMI in their classes enables them to improve their English literacy. This outcome can be readily explained by the fact that the constant use of a language will considerably improve the speaking, writing, reading, and listening abilities. This finding accords with Phuong and Nguyen’s (2019) outcomes, which indicated that instructing academic subjects through English results in increased English literacy. Moreover, many participants perceived increased academic interest to be one of the undeniable benefits of EMI courses. According to them, learning a new language can dramatically increase students’ interest in academic settings. This finding contradicts Yang et al.’s (2019) outcomes, which indicated that EMI students’ academic interests gradually decrease due to the unsatisfactory instruction. Furthermore, as the third important benefit of EMI classes, several participants referred to the role of EMI in familiarizing students with other cultures. They noted that studying academic subjects in English enables them to become familiar with the culture of English-speaking countries. It may be justified by the fact that cultural norms and values are the inseparable parts of a language (Kramsch, 2014; Sharifian, 2015; Yu, 2020). It implies that “those who truly learn a new language can enter into the cultural atmosphere in which that language exists” (Mazari and Derraz, 2015, p. 353). Additionally, some of the participants highlighted the crucial functions of EMI in expanding students’ English vocabulary, improving students’ academic knowledge, and activating students. These outcomes are consistent with those of some previous investigations (Goodman, 2014; Phuong and Nguyen, 2019; Drljaca and Vodopija-Krstanovic, 2020).

Likewise, as the findings of the thematic analysis demonstrated, the challenges of employing English in Chinese music classes can also be divided into six distinct themes: *more difficulty in comprehending course content*, *limited interactions between teachers and students*, *higher turnover intention among students*, *limited interactions among students*, *low classroom engagement*, and *low academic confidence*. It is worth noting that all the aforementioned challenges have something to do with EMI students’ limited English literacy. Regarding the first challenge, most students stated that using English as a means of instruction causes some serious problems for students in understanding the course content. It may be because students commonly attend EMI classes with limited English literacy. This outcome resonates with Pun and Thomas’ (2020) findings, which showed that EMI students often struggle with some serious linguistic challenges due to poor English language literacy. This finding also seems to be consistent with that of Kamaşak et al. (2021), who discovered that EMI students cannot easily understand classroom instructions as they do not have a good command of English. Concerning the second challenge, many participants articulated that using English as a medium of education reduces classroom interactions between students and teachers. This is in line with Ter-Vardanyan’s outcomes Ter-Vardanyan (2021), which revealed that EMI students are less likely to communicate with their teachers. As to the third challenge, many participants asserted that using English in music classes can dramatically increase the rate of turnover among students. This may be due to the unsatisfactory instruction that students receive in EMI courses. This outcome corroborates Yang et al.’s (2019) findings, which elucidated that EMI courses are commonly challenging for students due to the unsatisfactory and inappropriate instructions they receive in these courses. Regarding the rest of the problems of EMI classes, some of the Chinese music students mentioned that the employment of English in music classes causes limited interactions among students, low classroom engagement, and decreased academic confidence. It seems encouraging to compare these challenges with those reported in some previous studies (Aizawa and McKinley, 2020; Toh, 2020; Pun and Jin, 2021).

It should be mentioned that the outcomes of the current investigation are subject to three significant limitations. The most significant limitation lies in the fact that the present study’s data was gathered from a limited number of participants. As the inclusion of more participants can drastically increase the generalizability of the findings, future researchers are advised to perform their investigations with a large sample of EMI students. Another important limitation is that a pure qualitative method was adopted to carry out this research. To achieve more credible and comprehensive outcomes, mixed-method research is highly recommended. The last limitation of this study is that teachers’ views on EMI courses were disregarded. Put simply, only students were questioned about the benefits and challenges of EMI classes. Investigating EMI teachers’ viewpoints would help us to get a wider picture of the benefits and challenges of EMI classes. Future investigations should therefore concentrate on EMI teachers’ viewpoints.

## Conclusion and pedagogical implications

The current qualitative research was carried out with the aim of discovering the benefits and challenges of implementing EMI in Chinese music classes. As the outcomes of the thematic analysis revealed, improving students’ English literacy, enhancing students’ academic interests, and familiarizing students with other cultures were found to be the most important benefits of EMI courses. Moreover, difficulty in comprehending course content, limited interactions between teachers and students, and higher turnover intention among students were identified as the most serious challenges of EMI classes. With respect to the benefits and challenges raised by Chinese music students, it is possible to infer that EMI classes will be problematic and challenging only for those students who do not have a good command of English. Thus, the level of students’ English proficiency is highly critical in EMI courses. The outcomes of this inquiry appear to be enlightening and useful for those who are involved in Chinese EMI classes, including EMI teachers and students. It is because being aware of the potential challenges and benefits of EMI classes enables EMI teachers and students to make use of opportunities and minimize existing problems. Given the pivotal role that students’ English proficiency plays in EMI classes, students are required to read English books, listen to English podcasts, and attend English language classes in order to improve their English proficiency. In this respect, teachers are also expected to introduce some useful English resources to their students. In addition, the findings of this investigation may be illuminating for Chinese educational administrators and managers as well. The present study’s findings may help them capitalize on the significant opportunities of EMI classes and overcome the serious challenges of implementing EMI in Chinese music institutes.

## Data availability statement

The raw data supporting the conclusions of this article will be made available by the authors, without undue reservation.

## Ethics statement

The studies involving human participants were reviewed and approved by Shinawatra University Ethics Committee. The patients/participants provided their written informed consent to participate in this study.

## Author contributions

PS collected and analyzed the data and wrote the first draft of the manuscript. PS and JK reviewed and revised the manuscript. All authors contributed to the article and approved the submitted version.

## Conflict of interest

The authors declare that the research was conducted in the absence of any commercial or financial relationships that could be construed as a potential conflict of interest.

## Publisher’s note

All claims expressed in this article are solely those of the authors and do not necessarily represent those of their affiliated organizations, or those of the publisher, the editors and the reviewers. Any product that may be evaluated in this article, or claim that may be made by its manufacturer, is not guaranteed or endorsed by the publisher.

## Appendix A

**Open-ended scale**

**A Demographic information**
  Gender:
  Male.
  Female.
  Others (please specify).
  Prefer not to specify.
  If your answer to the above question is “Others,” it is optional to specify your gender here.
  Nationality:
  Age:

**Level of Education:**

**B Questions**
  1. What courses are you currently learning through English?
  Did you choose to learn these courses?
  If yes, why did you choose these English Medium courses?
  2. What do you think are the advantages of using English in music classes?
  3. What do you think are the disadvantages of using English in music classes?
  4. Do you have any difficulties when you learn courses in English?
  5. Do you have any further comments on the growing trend of English as a Medium of Instruction in China?
